# Pneumococcal Vaccine Breakthrough and Failure in Infants and Children: A Narrative Review

**DOI:** 10.3390/vaccines11121750

**Published:** 2023-11-24

**Authors:** Kristen Feemster, Jessica Weaver, Ulrike Buchwald, Natalie Banniettis, Kara S. Cox, E. David McIntosh, Vana Spoulou

**Affiliations:** 1 Merck & Co., Inc., Rahway, NJ 07065, USA; jessica.weaver@merck.com (J.W.); ulrike.buchwald@merck.com (U.B.); natalie.banniettis@merck.com (N.B.); kara_cox@merck.com (K.S.C.); 2 MSD (UK) Limited, London EC2M 6UR, UK; david.mcintosh1@msd.com; 3 Immunobiology and Vaccinology Research Laboratory, School of Medicine, National and Kapodistrian University of Athens, 11527 Athens, Greece; vspoulou@med.uoa.gr

**Keywords:** vaccine, breakthrough, failure, infants, children, pneumococcal, pneumonia

## Abstract

Globally, *Streptococcus pneumoniae* is a leading cause of vaccine-preventable morbidity and mortality in infants and children. In recent decades, large-scale pediatric immunization programs have substantially reduced the incidence of invasive pneumococcal disease. Despite this, residual vaccine-type pneumococcal disease remains in the form of vaccine breakthrough and vaccine failure. This targeted literature review aims to discuss aspects of vaccine breakthrough and failure in infants and children, including disease epidemiology, clinical presentation, risk factors, vaccination schedules, vaccine serotypes, correlates of protection, comorbidities, disease surveillance, and potential implications for future vaccine development.

## 1. Introduction

In the pediatric population, *Streptococcus pneumoniae* is a leading cause of pneumonia and acute otitis media (AOM) in children <5 years of age [1,2,3]. The bacterium can cause life-threatening invasive pneumococcal diseases (IPDs), such as meningitis and bacteremia, particularly in infants <1 year of age [4]. Over the past two decades, pneumococcal conjugate vaccines (PCVs) with increasing serotype coverage have been licensed for the prevention of pneumococcal disease (PD) in pediatric and adult populations [5,6] (Table 1).

**Table 1:** Pneumococcal vaccines approved for use in children >6 months of age globally ^†^.

Vaccine Type	Vaccine	Date of First Licensure	Serotypes Included
Pneumococcal polysaccharide vaccine (PPSV)	PPSV23 (Pneumovax 23) [7]	1983	1, 2, 3, 4, 5, 6B, 7F, 8, 9N, 9V, 10A, 11A, 12F, 14, 15B, 17F, 18C, 19F, 19A, 20, 22F, 23F, and 33F
Pneumococcal conjugate vaccine (PCV)	PCV7 (Prevnar) [8]	2000	4, 6B, 9V, 14, 18C, 19F, and 23F
	PCV10 (Synflorix) [9]	2009	1, 4, 5, 6B, 7F, 9V, 14, 18C, 19F, and 23F
	PCV10 (Pneumosil) [10,11]	2019 ^‡^	1, 5, 6A, 6B, 7F, 9V, 14, 19A, 19F, and 23F
	PCV13 (Prevnar 13) [6]	2009	1, 3, 4, 5, 6A, 6B, 7F, 9V, 14, 18C, 19A, 19F, and 23F
	PCV15 (Vaxneuvance) [12]	2021	1, 3, 4, 5, 6A, 6B, 7F, 9V, 14, 18C, 19A, 19F, 22F, 23F, and 33F
	PCV20 (Prevnar 20) [13]	2021	1, 3, 4, 5, 6A, 6B, 7F, 8, 9V, 10A, 11A, 12F, 14, 15B, 18C, 19A, 19F, 22F, 23F, and 33F

^†^ Includes pneumococcal vaccines approved in multiple countries globally. ^‡^ WHO pre-qualification date. PCV, pneumococcal conjugate vaccine; PCV7, 7-valent pneumococcal conjugate vaccine; PCV10, 10-valent pneumococcal conjugate vaccine; PCV13, 13-valent pneumococcal conjugate vaccine; PCV15, 15-valent pneumococcal conjugate vaccine; PCV20, 20-valent pneumococcal conjugate vaccine; PPSV, pneumococcal polysaccharide vaccine; PPSV23, 23-valent pneumococcal polysaccharide vaccine; WHO, World Health Organization.

A 7-valent PCV (PCV7; Prevnar™, Pfizer, New York, NY, USA) was the first to be licensed in 2000. In the same year, routine immunization with PCV7 was implemented in childhood vaccination programs in the United States (US), leading to a 99% decrease in the incidence of IPD caused by serotypes included in PCV7 [14,15]. Substantial reductions in PCV7 serotype-related disease have also been reported in many countries following the inclusion of PCV7 into regional childhood immunization programs [1,16].

Despite the overall reduction in disease incidence, an increase in IPD caused by non-PCV7 serotypes was subsequently observed [17], and higher-valency PCVs were developed to address this serotype replacement and increase overall coverage. A 13-valent PCV (PCV13; Prevnar 13™, Pfizer, New York, NY, USA), which eventually replaced PCV7, was licensed for use in infants and children ≥6 weeks of age by the European Medicines Agency (EMA) in 2009 and later by the US Food and Drug Administration (FDA) in 2010 [6,18]. PCV13 later became the first PCV to also be approved for use in adults [18]. More recently, a 15-valent PCV (V114; VAXNEUVANCE™, Merck Sharp & Dohme LLC, a subsidiary of Merck & Co., Inc., Rahway, NJ, USA) was approved in the US and the European Union (EU) for use in adults in 2021 and infants and children ≥6 weeks of age in 2022, respectively [12,19]. In 2021, a 20-valent PCV (PCV20; Prevnar 20™, Pfizer, New York, NY, USA) was approved for use in adults ≥18 years of age in the US and EU. Two years later, PCV20 was also approved in the US in infants ≥6 weeks of age, and it is currently under investigation for use in infants in the EU [13,20].

Owing to the large impact that PCVs have had on reducing PD in children, the World Health Organization (WHO) has recommended the inclusion of PCVs in national immunization programs. The effectiveness of PCVs has been demonstrated through the reductions in pediatric IPD, related hospitalizations, and deaths, as well as AOM, pneumonia, and nasopharyngeal carriage (NPC) caused by vaccine serotypes [1,16,21,22,23,24].

Large-scale immunization programs have both direct effects (on vaccinated individuals) and indirect effects (on unvaccinated individuals). Childhood PCV immunization programs have led to a reduction in the overall PD burden within older age groups through indirect community protection, attributed to a reduction in NPC and community transmission of vaccine serotypes. As such, the total impact on public health from PCV immunization programs is broad and multi-faceted [25].

Despite the great success of childhood PCV immunization programs, remaining PD is associated with significant morbidity and mortality in infants and children. Most of the residual PD is caused by non-vaccine serotypes. In addition, certain serotypes targeted by existing vaccines also continue to circulate and can cause residual vaccine-type (VT) disease, some of which occurs in vaccinated children. Childhood PCVs are typically administered as a two or three dose primary series up to 6 months of age, followed by a booster dose around 1 year of age. Vaccine breakthrough refers to PD caused by specific vaccine-preventable serotypes following partial vaccination (those who receive at least one dose of PCV but do not complete a full dosing schedule) [26,27]. Vaccine failure refers to PD occurring from a VT serotype after the completion of all planned vaccinations in a series, either through primary infant schedules (2 + 1, 3 + 1, or 3 + 0) or catch-up schedules (one, two, or three doses, based on age) [26].

Low rates of vaccine breakthrough or failure have been observed for cases of IPD in children, underscoring the high effectiveness of PCVs for most vaccine serotypes [26,28]. However, for those who experience vaccine breakthrough or failure, the burden of remaining PD caused by vaccine serotypes is associated with substantial morbidity or mortality. This burden is particularly prevalent in high-risk individuals, such as those with underlying diseases and immunodeficiencies, and remains a challenge for healthcare systems [26,29].

Previously published systematic reviews summarized the most frequently reported VT serotypes associated with vaccine breakthrough and failure, prior to, during, and following the introduction of PCV7, 10-valent PCV (PCV10), and PCV13 [26,30]. These reviews, which focus primarily on the epidemiology of IPD, are important in defining the VT serotypes responsible for residual IPD. However, available reviews have not included a comprehensive description of risk factors that also contribute to pneumococcal vaccine breakthrough and failure and, in turn, residual PD. In this narrative review of the literature we explore these factors, describing the clinical presentation of residual VT PD and discussing the potential implications for future pneumococcal vaccine development.

## 2. Vaccine Breakthrough and Failure in Infants and Children

### 2.1. Epidemiology of Vaccine Breakthrough and Failure in Infants and Children

In countries with a high uptake of PCVs, approximately 8% of the IPD cases observed in children ≤5 years of age, and 13% of IPD cases observed in children ≤17 years of age, are due to vaccine breakthrough or failure [26,28]. Throughout the PCV era, several serotypes have been associated with a large proportion of vaccine breakthrough and failure. Between January 2000 and April 2016, Oligbu et al. identified serotypes 19F and 6B as being responsible for more than two-thirds of cases of vaccine failure in countries with established PCV programs [30].

Similar results were observed in one systematic review that described the most commonly reported serotypes in PCV10 or PCV13 breakthrough infections across 26 studies. In studies of PCV10, serotypes 14, 6B, and vaccine-related serotypes 19A and 6A (cross-reactive with 6B and 19F) were the serotypes most responsible for vaccine breakthrough and failure. In studies of PCV13, serotypes 3, 19A, and 19F were responsible for 40.0%, 35.9%, and 14.3% of vaccine failures, respectively [26]. Following the introduction of PCV13, one trial demonstrated that serotype 19A accounted for 81.8% of vaccine failures or breakthroughs [31]. In addition, another study identified serotypes 3, 7F, and 19A as being the most commonly related to vaccine failures [28]. Similarly, an Australian study found that serotype 3 accounted for 76.4% of childhood pneumococcal empyema, with 97.1% of children having received at least one dose of a pneumococcal vaccine [32].

Differences in the frequency of certain serotypes as a cause of breakthrough disease and vaccine failure may be related to differences in the number of doses and vaccination schedules in different countries, as well as the local epidemiology of PD before the introduction of PCVs in national immunization programs.

### 2.2. Clinical Presentation of PD after a Breakthrough or Failure in Vaccinated Children

Clinical presentation of PD as a consequence of vaccine breakthrough or failure in children may vary. Data from a study by Oligbu et al. indicated that children with PCV13 vaccine serotype failure were more likely to be healthy and develop a lower respiratory tract infection than those who experienced PCV7 vaccine serotype failure [33]. Higher rates of PCV13 vaccine failure, often presenting with pneumonia, have been observed in older children (>2 years of age) compared with those of a younger age [28].

Cases of vaccine failure are mostly caused by serotypes that are more virulent or resistant to antibiotics (3 and 19A) [26,34,35]. From this, it could be predicted that cases of vaccine breakthrough and failure associated with these serotypes may exhibit increased severity compared with cases linked to other serotypes. One study investigated disease manifestations in children vaccinated with PCVs and found that children who experienced vaccine failure had a higher likelihood of complicated pneumonia than children who did not experience vaccine failure (odds ratio (OR) 6.65; 95% confidence interval (CI): 1.91–23.21), the majority of which were cases of pneumonia with empyema [36].

However, one caveat to note is that the severity of breakthrough disease is not well described in these reports, and information regarding treatment, hospitalization, and mortality rates is lacking. Therefore, it is difficult to draw conclusions on any potential differences in the disease severity between pneumococcal breakthrough disease and disease in unvaccinated children.

### 2.3. Vaccine Schedules and Incomplete Vaccination

Breakthrough disease and vaccine failure in children were analyzed in a systematic review assessing IPD incidence in partially or fully vaccinated children between 2008 and 2019 [26].

During the study period, the rates of VT PCV breakthrough and failure were 9.3% and 8.4% of all IPD cases in vaccinated children, respectively. One factor affecting the incidence of vaccine failure and breakthrough was regional differences in dosing schedules (e.g., failures were more common following a 3 + 0 dosing schedule than a 2 + 1 or 3 + 1 schedule) [26]. However, when assessing the impact of a booster dose on vaccine failures, there were no significant differences observed in the rates of IPD in children receiving 2 + 1 compared with 3 + 1 schedules during any follow-up period after series completion [26].

Age at the time of vaccine breakthrough or failure was not reported in most studies included in the analysis, but authors suggested that a substantial number of vaccine failures likely occurred up to many months or years after the last dose, due to waning immunity [26].

One study assessing breakthrough disease after PCV7 and PCV13 introduction found that a schedule with three primary PCV7 doses resulted in fewer cases of breakthrough disease compared with a two-dose schedule in the first year of life (incidence rate (IR): 7.0 (2 + 0) versus 0.3 (3 + 0); IR ratio: 21.8; 95% CI: 5.3–89.3). Similarly, for PCV13, a three-dose primary schedule was associated with statistically significantly fewer breakthrough infections than a two-dose primary schedule (2 + 0) (IR: 7.8 (2 + 0) versus 0.6 (3 + 0); IR ratio: 12.9; 95% CI: 4.1–40.4). Most breakthrough infections associated with both vaccines were observed within the first year of life, with a similar frequency of infections observed across the age groups 0–5 months of age (PCV7, 14.0%; PCV13, 11.7%) and 6–11 months of age (PCV7, 17.4%; PCV13, 18.9%) [27].

There is increasing evidence that the inclusion of booster doses in infant immunization schedules has a major impact on reducing the risk of vaccine failure. A study by Yildrim et al. investigated the effectiveness of PCV13 over a 7-year period and demonstrated that the likelihood of vaccine failure was lowest among children who had received three primary doses plus one booster dose (3 + 1), compared with alternative dosing schedules, including with and without a booster [28]. However, as previously mentioned, significant differences in reductions in IPD through the administration of booster doses have not been observed, particularly when comparing 2 + 1 and 3 + 1 dosing schedules [26].

Recent updates to the recommended dosing schedule in Australia reinforce the importance of a booster dose. The dosing schedule for PCV13 infant immunization was updated from a 3 + 0 schedule, given at 0, 4, and 6 months of age, to a 2 + 1 schedule, administered at 2, 4, and 12 months of age [37]. This update came as a result of an increasing incidence of IPD in fully vaccinated children and a subsequent review of effective dosing schedules. Data from three studies demonstrated that a three-dose primary schedule without a booster dose was associated with antibody levels below the correlates of protection against IPD [32,37,38]. The Australian Technical Advisory Group on Immunization (ATAGI) compared IPD surveillance data in Australia with those from the United Kingdom (UK) and found that the IPD vaccine failure rate in children ≥12 months of age was substantially higher in Australian children vaccinated with a 3 + 0 dosing schedule than with children in the UK given a 2 + 1 dosing schedule (3.4 vs. 0.7 per 100,000 person-years) [37]. Considering the evidence, the ATAGI proposed a change to adopt the 2 + 1 dosing schedule, which was implemented in 2018 and is expected to improve direct protection from the PCV13 infant immunization program [38].

### 2.4. Correlates of Protection Associated with Vaccine Breakthrough and Failure

Well-conducted efficacy trials of PCV7 in infants and toddlers have enabled the evaluation of correlates of protection against IPD, based on pooled immunogenicity data. An immunoglobulin G (IgG) antibody concentration of 0.35 μg/mL measured after the third dose of a primary series was recommended as a threshold value for correlates of protection for anti-capsular antibodies against IPD in infants. These recommendations have become the criterion recommended by the WHO for the licensure of new PCVs [39,40]. However, it is now understood that such thresholds may represent a relative degree of protection rather than an absolute one and that serotype-specific correlates of protection or NPC vary between serotypes and disease manifestations (e.g., IPD, AOM) [41]. A study by Andrews et al. assessed the effectiveness and immunogenicity of PCV7 and PCV13 in infants and demonstrated that the vaccine effectiveness for both vaccines was lower than predicted by the aggregate correlate of protection of 0.35 μg/mL used during licensing. Serotype-specific correlates of protection for IPD were higher than 0.35 μg/mL for serotypes 1, 3, 7F, 19A, and 19F and lower than 0.35 μg/mL for serotypes 6A, 6B, 18C, and 23F. The calculated serotype-specific correlates of protection for enzyme-linked immunosorbent assay (ELISA) were as follows: serotype 1 (0.78 μg/mL), 3 (2.83 μg/mL), 6A (0.16 μg/mL), 6B (0.16 μg/mL), 7F (0.87 μg/mL), 18C (0.14 μg/mL), 19A (1.00 μg/mL), 19F (1.17 μg/mL), and 23F (0.20 μg/mL). There may be implications for an increased risk of breakthrough disease and vaccine failure for serotypes that require higher levels of antibodies for protection against disease [42]. It is possible that some serotypes may require more vaccine doses than others to reach a protective titer. One study revealed that serotypes 6B and 23F required at least three primary infant doses to produce a response over the 0.35 µg/mL correlate of the protection threshold [43].

When considering vaccine effectiveness against NPC and mucosal disease, such as AOM, serotype-specific correlates of protection may also vary and require even higher antibody levels than those required for protection against IPD. One study investigated the impact of PCV13 on NPC and AOM in infants and observed variation in the levels of serum antibodies required for protection, with serotypes 22F, 6A, and 19A requiring serum antibody levels of 0.45 μg/mL, 0.51 μg/mL, and 4.1 μg/mL to prevent NPC, respectively. Correlates of protection required for the prevention of AOM were 0.25 μg/mL for serotypes 22F, 33F, and 6A and 2 μg/mL for serotype 19A [44]. These results may also have implications for the indirect protection provided by PCVs through a reduction in NPC. If higher levels of antibodies for certain serotypes are required to prevent carriage, vaccination with existing PCVs that induce lower serotype-specific antibodies than the retrospective correlates of protection may result in increased rates of NPC, which could, in turn, contribute to persistent community transmission [44].

An additional factor to consider when discussing correlates of protection is the development of higher-valency PCVs. Increasing serotype valency in newly developed PCVs can lead to decreased levels of immunogenicity, which may impact vaccine effectiveness and, in turn, affect the rates of NPC and mucosal disease [45,46]. One study compared the proportion of infants achieving serotype-specific concentrations of ≥1.0 µg/mL in those vaccinated with PCV10 or PCV13. There was a significantly higher proportion of infants achieving the threshold for serotypes 6B, 18C, and 19F in the PCV10 group compared with the PCV13 group. However, the rates of NPC at 9 months of age were similar between both vaccine groups, suggesting that there are limitations in using vaccine immunogenicity as a sole predictor of vaccine effectiveness against NPC and breakthrough disease [45]. It is important to note that it may not be accurate to extend this conclusion to all serotypes included in higher-valency vaccines that have recently been developed (PCV20). Since vaccine effectiveness data are not yet available, further research will be required to fully explore the impact of higher valency on NPC in infants and children.

### 2.5. Comorbidities

In addition to vaccine schedules and serotype-specific correlates of protection, the risk of breakthrough disease and vaccine failure has also been associated with the presence of comorbid conditions, such as immunodeficiencies, malignancies, and several chronic diseases. A high prevalence of comorbidities has been identified among cases of vaccine failure in children ≤5 years of age. These include multiple immunocompromising conditions, such as human immunodeficiency virus (HIV), nephrotic syndrome, and sickle cell disease, as well as chronic lung or heart disease [28,30,47]. A study in England and Wales explored vaccine failure after PCV7 and PCV13 vaccination in children. They found that approximately one-third of PCV13 failure cases were associated with a comorbidity, primarily immunosuppression and cardiac abnormalities [33]. In addition, a systematic review reported that children with certain underlying comorbidities (e.g., prematurity or immunocompromising conditions) are at a higher risk of developing IPD, regardless of vaccination status [48].

Studies have also evaluated the impact of specific comorbidities on IPD. In children with sickle cell disease, a systematic review reported that ~1.9% of the population experienced IPD in the era of PCVs. The majority of IPD identified in children was due to non-PCV13 serotypes. The results demonstrated that children with sickle cell disease remain at a higher risk of IPD than healthy peers without sickle cell disease [49].

A systematic review of 10 studies estimated the effectiveness of infant PCV vaccination among children with HIV compared with children without HIV. The studies included different dosing schedules, including 3 + 0, 2 + 1, and 3 + 1. Nine of the studies were conducted in South Africa, and one was conducted in the US. In children <7 years of age, results from randomized trials showed that the overall effectiveness of PCVs in preventing VT IPD among children with HIV was substantially lower than among children without HIV (51.0% vs. 77.3%, respectively). These results demonstrate the higher risk of IPD and vaccine failure in children with HIV [50].

Although PCV breakthrough disease and vaccine failure are relatively rare, these studies highlight the overall susceptibility to IPD and severe disease in children with comorbidities as a result of vaccine breakthrough or failure, as well as non-VT disease.

### 2.6. Discussion

This review suggests that the risk of breakthrough disease and vaccine failure is higher for certain serotypes and is associated with receipt of fewer primary infant PCV doses, which may impact the likelihood of achieving protective thresholds. The ability to achieve such thresholds is further mediated by the immunogenicity of the vaccine administered and the child’s immune response [27,51,52]. Comorbidities, such as immunodeficiencies and cardiac abnormalities, may affect the host response and, thus, increase the risk of breakthrough disease [33] (Table 2). The impact of other respiratory viruses on NPC and vaccine breakthrough or failure is not well elucidated. There is some evidence to suggest that there is an increased risk of contracting pneumococcal disease in the presence of coinfection with either influenza or respiratory syncytial virus [53,54,55]. Despite this, the impact of co-infection on vaccine effectiveness, and consequently on vaccine breakthrough or failure, has not been described, and further investigation is warranted.

## 3. Vaccine Breakthrough and Failure: Implications for Surveillance and Future Vaccine Development

Surveillance of pneumococcal serotype prevalence is imperative to assess the impact of PCVs and monitor trends in breakthrough disease and vaccine failure, particularly as new PCVs are introduced. The Pneumococcal Serotype Replacement and Distribution Estimation (PSERENADE) project aimed to assess the global burden of PD and estimate the impact of PCV10 and PCV13 by age, product, schedule, and disease. While this study found limited surveillance data from high-burden, low-income countries, it provided a sizeable and representative dataset that will be able to inform researchers and policymakers [56].

The goal of surveillance is to characterize national and local epidemiologic trends, detect geographic and temporal changes, monitor the impact of vaccines on disease incidence, and inform future vaccine development. However, the availability of robust surveillance infrastructure is inconsistent [4]. Across laboratories, there is limited serotyping capacity, as the methods required to identify serotypes are not widely available [57]. In addition, diagnostic methods are limited for non-invasive disease and necessitate alternative approaches [58].

Increasing the serotyping capacity of laboratories is one way to improve surveillance. Traditional serotyping techniques, such as the gold-standard Quellung reaction, are laborious and require technical training; thus, they are not widely available in most clinical laboratories [57]. In addition, to employ this technique, pneumococcus must be isolated via culture to identify a pneumococcal serotype. However, a typical yield of microbiological diagnostics is as low as 8% and <10% for pleural fluid or blood, respectively [59]. Previous treatment with antibiotics in children may be one of the contributing factors to this low yield. For example, serotype 3 is infrequently cultured from samples using traditional techniques but is often detected by PCR-based assays [60,61]. As a result, the lack of comprehensive data using traditional serotyping techniques hinders our ability to fully assess the serotypes involved in vaccine breakthrough and failure.

The use of polymerase chain reaction (PCR) to identify the genes responsible for individual capsular serotypes may be an alternative approach that is feasible for some state public health centers [4]. PCR testing has high specificity and sensitivity, and can provide accurate readouts with a very low rate of false negatives [62]. Following the severe acute respiratory syndrome coronavirus 2 (SARS-CoV-2) pandemic, PCR infrastructure is more established and could potentially be leveraged for this purpose [63].

In addition, novel serotype-specific assays have been developed with the aim of detecting cases of non-invasive PD, such as pneumonia, commonly missed by traditional testing methods. Serotype-specific urinary antigen detection (SSUAD) assays have been developed to detect the 13 serotypes included in PCV13 or the 15 serotypes included in V114 [58,64]. Early studies using SSUAD demonstrated increased detection of pneumococcal pneumonia among adults hospitalized with community-acquired pneumonia and IPD, although their utility in pediatric populations has not yet been explored [58,65].

Accurate reporting of vaccination status and comorbidities is also important when evaluating epidemiologic trends in breakthrough disease and vaccine failure in children, yet these data are not routinely recorded for PD cases. This gap in accurate reporting is particularly common in low- to middle-income countries, where the systems to maintain medical records and immunization registries may not be well established [66].

To better evaluate the potential effectiveness of current and newly developed pneumococcal vaccines, there is also a need to identify more effective and reliable immunological correlates of serotype-specific protection. This will help to improve the evaluation of vaccine efficacy and effectiveness for new PCVs for which immunogenicity is the primary available clinical endpoint. Some progress has been made in this regard, whereby modeling was used to predict the effectiveness of the recently approved 15-valent PCV, V114, against IPD in children. In that study, protective antibody concentrations were estimated for each of the individual serotypes. The results suggest that a high overall benefit would be gained from increased serotype 3 effectiveness, and predicted levels of protection against other serotypes would be maintained [67]. Such considerations should aid in the design and development of future PCVs, in which epidemiologically important serotypes to be included in higher-valency vaccines are sufficiently immunogenic in new formulations.

Lastly, given the association between incomplete vaccination and breakthrough PD, it is important to find ways to increase compliance with vaccine schedules. Approximately 79% of infants vaccinated with PCV13 in the US born in the years preceding the SARS-CoV-2 pandemic (2017–2019) completed the series, leaving a substantial number only partially vaccinated [68]. Similarly, in the WHO European region, estimates of vaccine series completion rates averaged at 82% among children ≤1 year of age in 2021 [69]. Vaccine equity is an important driver for increasing both access to primary immunization and compliance with vaccine schedules. Factors such as age, ethnicity, location (urban or rural), and socioeconomic status have contributed to differences in childhood immunization coverage across a region [70]. Vaccine inequalities exist not just for overall coverage but also for the completion of vaccine schedules and timing of doses. Importantly, disparities in timely series completion can lead to disparities in breakthrough disease and overall pneumococcal disease risk. Therefore efforts to develop frameworks that address inequalities within vaccination programs are paramount, in order to improve vaccine uptake and address the community, institutional, and policy factors that threaten vaccine equity [70].

## 4. Conclusions

Despite the success of pneumococcal vaccination programs, vaccine breakthrough and failure still occur in the pediatric population, including the most vulnerable, contributing to a substantial global burden due to high morbidity and mortality in young infants and children. Current evidence demonstrates that incomplete dosing schedules, reduced serotype-specific vaccine effectiveness (most notably in serotypes 3, 6A, and 19A) and the presence of comorbid conditions are important factors associated with the development of breakthrough disease and vaccine failure in children. Improved characterization of vaccine breakthrough and failure in a clinical setting, paired with the identification of disease-causing serotypes, an accurate assessment of vaccination status and the presence of any associated comorbidities, will contribute to a more robust evaluation of potential associations between these variables and the incidence of breakthrough disease.

This, in turn, will provide a foundation of evidence to support the development of newer vaccines that maintain or improve on immunogenicity for serotypes commonly associated with breakthrough infections. This evidence will also help to inform dosing schedules and indications for certain at-risk populations.

## Figures and Tables

**Table 2:** Factors associated with pneumococcal vaccine breakthrough and failure in infants and children.

Factor(s)	Commentary	Supporting Literature
Dosing schedule	Excluding booster (toddler) doses, a lower number of primary infant doses with PCV13 or PCV7 is associated with higher vaccine breakthrough and failure. Once a booster (toddler) dose is given, the rates of vaccine breakthrough and failure decrease with both PCV13 and PCV7.	Mungall et al., 2022 [26] Adebanjo et al., 2020 [27] Yildirim et al., 2020 [28] Zimmerman et al., 2019 [38] Blyth et al., 2020 [37]
Comorbidities	Underlying comorbidities, such as immunodeficiencies and cardiac abnormalities, have been associated with a higher incidence of vaccine breakthrough and failure, particularly with lower-valency vaccines, such as PCV7.	Oligbu et al., 2017 [33] Hsu et al., 2005 [47] Oligbu et al., 2016 [30] Oligbu et al., 2019 [49] Yildirim et al., 2020 [28] Yildirim et al., 2015 [48] Vardanjani et al., 2019 [50]
Correlates of protection and serotype-specific effectiveness	PCV13 serotypes 3, 7F, 19A, and 19F are the most commonly reported in vaccine breakthrough and failure, and antibody levels required for sufficient protection against these serotypes are higher	Mungall et al., 2022 [26] Ricketson et al., 2022 [52] Almeida et al., 2016 [31] Andrews et al., 2014 [42] Kaur et al., 2021 [44] Oligbu et al., 2016 [30] Spijkerman et al., 2013 [43] Conklin et al., 2014 [51] Yildirim et al., 2020 [28] Strachan et al., 2021 [32]

PCV7, 7-valent pneumococcal conjugate vaccine; PCV13, 13-valent pneumococcal conjugate vaccine.

## Data Availability

No new data were created or analyzed in this study. Data are contained within the article.
